# Which people with diabetes are treated with a disposable, half‐unit insulin pen? A real‐world, retrospective, database study in Spain

**DOI:** 10.1002/edm2.451

**Published:** 2023-09-15

**Authors:** F. Javier Ampudia‐Blasco, Natalia Duque, Esther Artime, Elena Caveda, Erik Spaepen, Silvia Díaz‐Cerezo, Miriam Rubio‐ de Santos, Daniel Callejo Velasco, M. Pilar Bahíllo‐Curieses

**Affiliations:** ^1^ Endocrinology and Nutrition Department Clinic University Hospital Valencia, INCLIVA Research Foundation Valencia Spain; ^2^ Eli Lilly and Company Madrid Spain; ^3^ HaaPACS GmbH Schriesheim Germany; ^4^ IQVIA Madrid Spain; ^5^ Servicio de Pediatría, Endocrinología Pediátrica, Hospital Clínico Universitario de Valladolid Valladolid Spain

**Keywords:** diabetes mellitus, Type 1, diabetes mellitus, Type 2, insulin lispro, retrospective study

## Abstract

**Introduction:**

Insulin lispro 100 units/mL Jr KwikPen is the first prefilled, disposable, half‐unit insulin pen that delivers 0.5–30 units in increments of 0.5 units for the treatment of patients with diabetes. This study describes the profile of patients in Spain who initiated insulin therapy with Jr KwikPen in a real‐world setting.

**Methods:**

This retrospective, observational study based on IQVIA's electronic medical records database included patients with Type 1 (T1D) or Type 2 (T2D) diabetes who initiated therapy with Jr KwikPen between May 2018 and December 2020. Sociodemographic, clinical, and treatment characteristics at treatment initiation were analysed descriptively.

**Results:**

A total of 416 patients were included. The main characteristics of the T1D/T2D groups (*N* = 326/90), respectively were as follows: female sex, 61.7%/65.6%; mean age (standard deviation [SD]), 32.5 (20.7)/55.5 (16.6) years; body mass index, 20.9 (4.2)/25.2 (4.6) kg/m^2^ (*N* = 239/77); HbA1c, 7.8 (1.7)%/8.0 (1.5)% (*N* = 141/64); and presence of diabetes‐associated comorbidities, 27.9%/64.4%. Only 32.8% of patients with T1D were < 18 years old. Among Jr KwikPen users, 12.3% (T1D/T2D, 7.7%/28.9%) were ≥ 65 years old, 17.1% patients were newly diagnosed, and 3.8% were pregnant women. The mean (SD) total insulin dose pre‐index for T1D/T2D was 43.1 (23.6) and 40.7 (21.6) UI/day, respectively. The mean (SD) insulin dose at the start of Jr KwikPen use was 26.63 (16.56) and 22.58 (13.59) UI/day for T1D/T2D, respectively. Jr KwikPen was first prescribed mainly by endocrinologists (58.7%) or paediatricians (22.6%).

**Conclusions:**

The profile of patients who initiated therapy with Jr KwikPen in routine practice was broad with many patients being adults. Most of these patients had T1D, inadequate glycemic control, and multiple associated comorbidities. These results suggest that Jr KwikPen is prescribed in patients who may benefit from finer insulin dose adjustments, namely children, adolescents, adults, older individuals, or pregnant women with T1D or T2D.

## Graphical abstract

Insulin lispro 100 units/mL Jr KwikPen® is the first insulin lispro 100 units/mL prefilled, disposable, half‐unit insulin pen that delivers 0.5–30 units in steps of 0.5 units for the treatment of Type 1 diabetes (T1D) and Type 2 diabetes (T2D). This study used electronic medical records to study the profile of patients with T1D and T2D in Spain who initiated therapy with Jr KwikPen between May 2018, when this drug was made commercially available, and December 2020. Findings from this study highlights that a broad range of patients used the Jr KwikPen device in routine practice, such as children, adolescents, adults, elderly people and pregnant women.

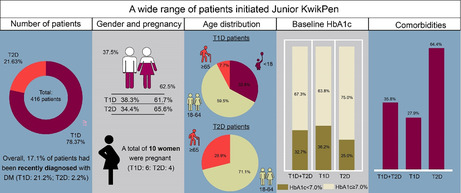

## 1 INTRODUCTION

Diabetes mellitus is a major public health concern worldwide because of its increasing prevalence, contribution to the development of chronic diabetes‐related complications and associated high mortality. A total of 1,211,000 children and adolescents younger than 20 years are diagnosed with Type 1 diabetes (T1D) globally, and about 108,000 new cases were diagnosed in children under the age of 15 years in 2021.^1^ The prevalence of Type 2 diabetes (T2D) increases with age and is higher in elderly populations.^2^ In 2021, the International Diabetes Federation estimated that over 500 million people worldwide and 5,141,000 people in Spain between the ages of 20 and 79 years were living with diabetes.^1^ The high prevalence of diabetes has a significant economic impact on healthcare systems worldwide. In Spain, the total direct annual cost of diabetes was estimated at €5,809,000,000, representing 8.2% of the total Spanish health expenditure.^3^

Insulin treatment to achieve blood glucose levels close to the normal range is the mainstay of therapy for T1D.^4^ This is achieved with multiple daily injections of rapid and long‐acting insulin or with continuous subcutaneous insulin infusion. For T2D, guidelines recommend that patients be started on pharmacologic treatment with metformin and that treatment be intensified as the disease progresses or treatment fails to achieve or sustain glycemic goals.^5^ Many patients with T2D eventually require insulin therapy. Glycemic targets should be individualized considering factors including duration of diabetes, age and life expectancy, comorbid conditions, known cardiovascular disease or microvascular complications and impaired awareness of hypoglycemia.^4^

Glycemic targets are particularly challenging to attain for adolescents and young adults^6^, ^7^, ^8^ as well as for elderly patients.^9^ Young children typically require smaller insulin doses because of their lower body weight and greater sensitivity to insulin, while elderly patients with diabetes are generally at a notably higher risk for severe hypoglycemia and its complications because of age, duration of diabetes and greater prevalence of hypoglycemia unawareness.^10^, ^11^, ^12^, ^13^ This risk increases when cognitive and/or physical impairments and other comorbidities are present, as they affect insulin requirements and reduce insulin clearance, which result in increased insulin sensitivity. Precision dosing is especially critical for these patients, as they are most susceptible to insulin dosing mismatch.^10^, ^14^ Pregnant women with diabetes are also a subpopulation with stringent glucose targets because of pregnancy‐related complications.^15^ Pregnancy affects glycemic control profoundly, with increased glucose variability and an increased risk of hypoglycemia.^16^, ^17^ Athletes may also benefit from precision dosing to aid in glycemic control when varied carbohydrate intake, differences in exercise and non‐exercise days, and risk of hypoglycemia are taken into account.^18^, ^19^

Half‐unit insulin pens deliver insulin in 0.5 U increments. This extends the well‐known benefits of insulin pens to allow precise dosing for mealtimes and physical activities even in erratic diet situations or health conditions through delivery of corrective doses in small increments. In this context, half‐unit pens are ideal for populations who require lower doses of insulin or patients who are insulin‐sensitive and require greater accuracy and precision in insulin delivery to achieve more stringent glycemic control.^20^ Several half‐unit pens have been launched in recent years: HumaPen® Luxura HD™ (Eli Lilly and Company; injects 0.5–30 units of insulin from a 3 mL cartridge (100 IU/mL) one half unit at a time),^21^ InPen™ (Medtronic Diabetes; a reusable, smart insulin pen that syncs to a mobile app),^22^ NovoPen Echo® (Novo Nordisk; specifically designed for the paediatric population and combines half‐unit dosing with a simple memory function),^23^ JuniorSTAR® (Sanofi Diabetes; injects 1–30 units of insulin one half unit at a time),^24^ and Humalog® Junior KwikPen® (Eli Lilly and Company), with the last three being launched in the Spanish market. The extensive use of interstitial glucose monitoring (flash or continuous) allows more precise dose adjustments and with them, the use of half‐unit pens becomes important.^9^

Insulin lispro 100 units/mL Junior KwikPen (Jr KwikPen), commercially available in the Spanish market since 2018, is the first available insulin lispro 100 units/mL prefilled, disposable, half‐unit insulin pen that delivers 0.5–30 units in steps of 0.5 units.^25^ This offers tighter insulin dose adjustments than its integer‐unit counterparts (i.e. Humalog 100 KwikPen), thus providing precision to the insulin regimen and allowing insulin‐sensitive populations to achieve tight glycemic control. The insulin in Jr KwikPen is a rapid‐acting insulin indicated for the treatment of adults and children with diabetes who need rapid‐acting insulin in addition to long‐acting insulin for the maintenance of normal glucose levels as well as for the initial stabilization of diabetes.^26^, ^27^, ^28^

The primary objective of this study was to describe the sociodemographic, clinical and treatment characteristics of patients with diabetes who initiated treatment with Jr KwikPen in real‐world practice in Spain and to answer the question ‘which groups of people with diabetes are treated with Junior KwikPen in the real‐world?’

## 2 MATERIALS AND METHODS

This was a retrospective, observational study conducted using the IQVIA's electronic medical records (EMRs) database in Spain. IQVIA's EMRs database is an existing national, longitudinal, primary and secondary care database that collects anonymized patient data since 2006. It contains anonymized EMRs of patients collected through a constant panel of over 3000 office‐based primary and secondary care physicians who are equipped with IQVIA's EMR software. The active patients in the database represent around 3% of the Spanish population. Longitudinal patient data are uploaded and delivered to IQVIA monthly, through an electronic link containing the full anonymized data of every patient in participating practices (Supporting Information S1).

The study was approved by the accredited Clinical Research Ethics Committees of the Hospital Clínic de Barcelona before study initiation. The study was also conducted according to Good Clinical Practice guidelines (International Conference of Harmonization) and the Declaration of Helsinki.

### 2.1 Study design and population

**FIGURE 1:**
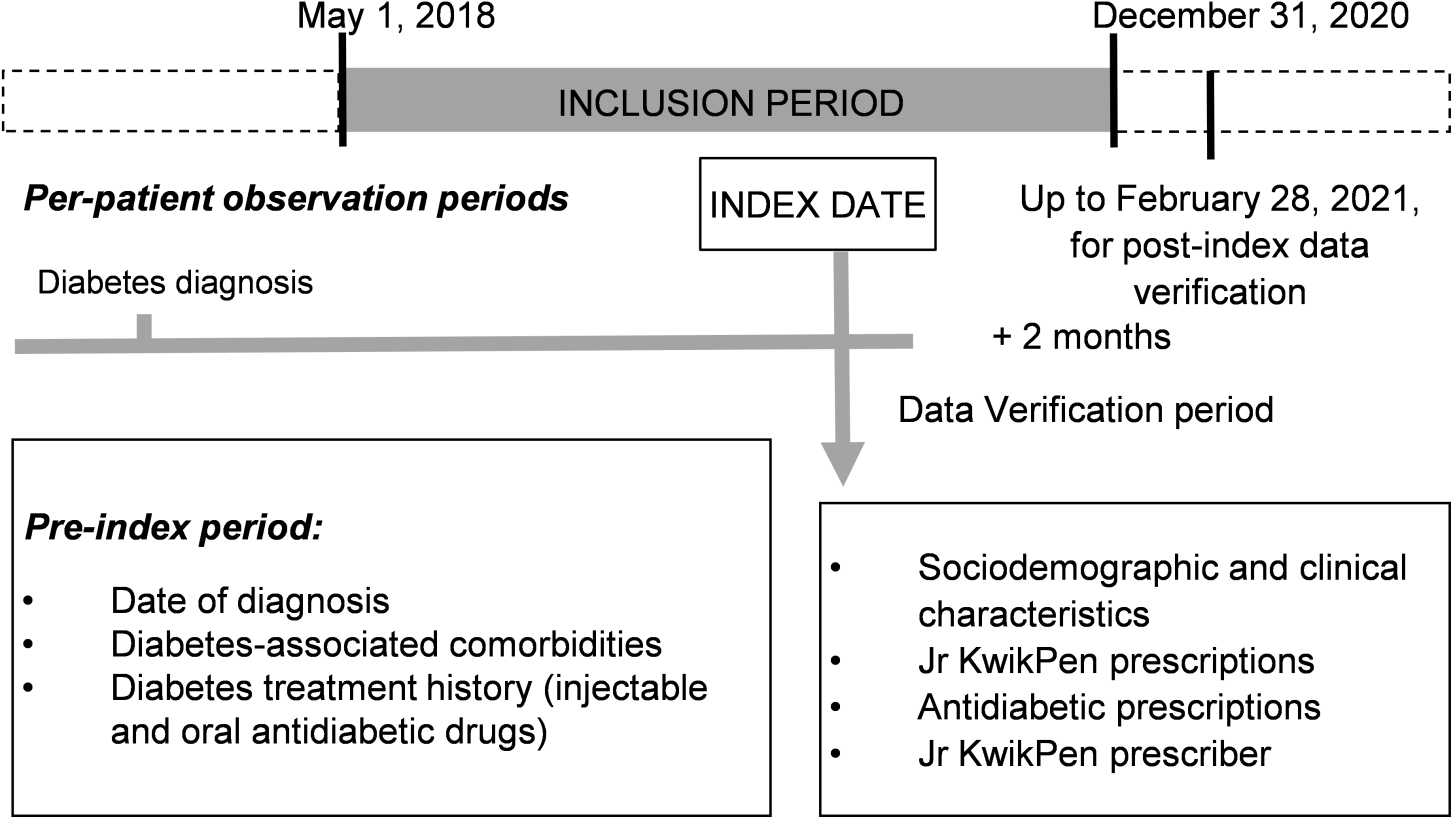
Study design. Per‐patient observation periods extended from diabetes diagnosis to last data available prior to the index date. Jr KwikPen, Junior KwikPen.

Patients of all ages with T1D, T2D, or gestational diabetes who started treatment with Jr KwikPen between 1 May 2018, (launch of Jr KwikPen in Spain) and 31 December 2020, were eligible for inclusion in the study (Figure 1). For each patient, the index date was defined as the start date of treatment with Jr KwikPen, between 1 May 2018, and 31 December 2020. All information from patients exposed to the treatment of interest was obtained from IQVIA's EMRs database.

The presence of pregnancy and frailty at the index date was explored using proxies in the database and published algorithms where possible. Pregnancy was identified based on the presence of the ICD9‐CM diagnostic codes for pregnancy (Table S1). For this subgroup, three definitions of pregnancy were considered: a first pregnancy within the 3 months before the index date (for patients with a gestational diabetes diagnosis, a time frame of 6 months before the index date was to be considered); a first pregnancy or gestational diabetes diagnosis during treatment with Jr KwikPen (period between index date and 2 months after); and a pregnancy or gestational diabetes diagnosis during treatment with Jr KwikPen, with no other pregnancy recorded in the previous 12 months.

Frailty was defined using the FRAIL scale, composed of five components or frail items (Fatigue, Resistance, Ambulation, Illness and Loss of weight), which was previously used for diabetes.^29^, ^30^ The assessment of each frail item, except ‘Illnesses’, was based on the presence of any of the selective diagnoses of each component (ICD‐9 CM diagnostic code) in the patient's medical data (Table S1) within the 5 years preceding the index date. The ‘Illnesses’ item was based on the presence of at least four out of the five morbidities listed. Patients were categorized into non‐frailty (0 frail components), pre‐frailty (presence of one or two frail components) and frailty (presence of more than two frail components) groups.

### 2.2 Statistical analysis

Descriptive analyses were performed to obtain frequency and proportion for categorical variables, and mean, median, standard deviation (SD), 25th and 75th percentiles, minimum, and maximum for continuous variables. The description of missing data for each outcome of interest is provided (e.g. *N* total of subjects, n subjects with data, *n* missing). Missing values were not imputed. IQVIA's EMRs database only contains data from 2008 onward, which limited the availability of data from the past (diagnosis date, prior treatments, etc.). Therefore, certain assumptions had to be made for the purpose of the analysis, for example, the date of diagnosis for patients diagnosed before 2008 was imputed to the date of the first record available in IQVIA's EMRs database and is referred to as the time from first diabetes record.

Analyses are presented overall and by type of diabetes (T1D and T2D) and age group: <18, 18–64 and ≥ 65 years for patients with T1D and 18–64 and ≥ 65 years for patients with T2D. The statistical analysis was performed by statisticians from IQVIA, using SAS Enterprise Guide Version 7.15.

## 3 RESULTS

### 3.1 Patient sociodemographic and clinical characteristics

**FIGURE 2:**
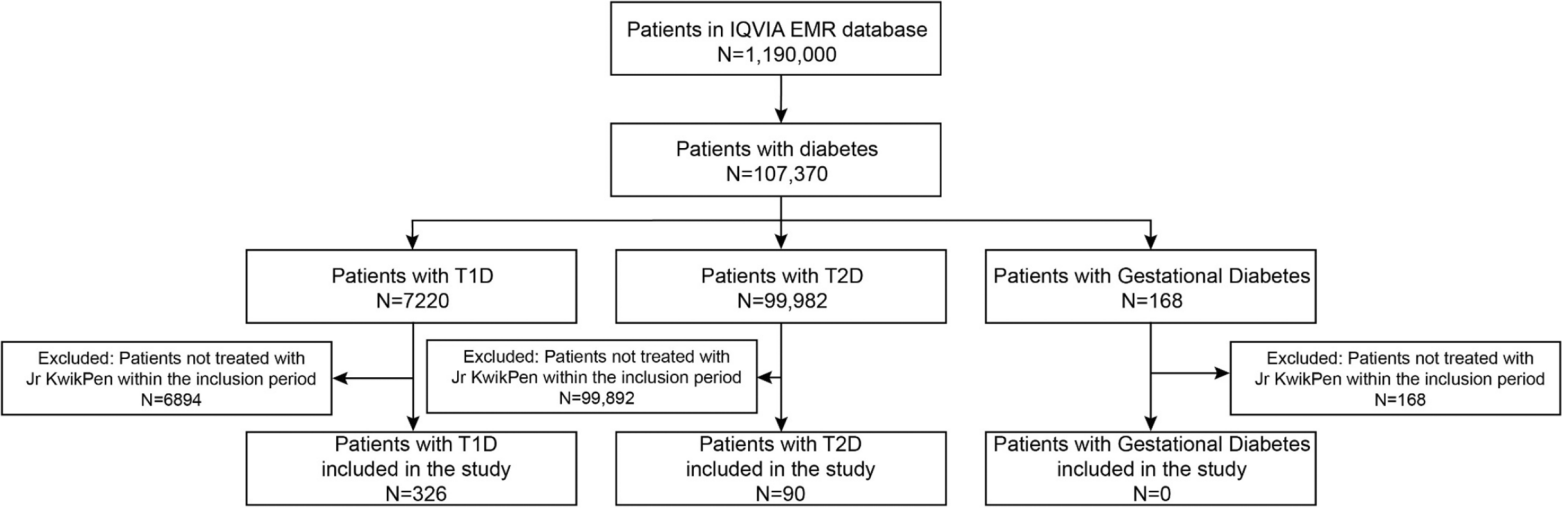
Patient disposition. Gestational diabetes was investigated, but no patients with gestational diabetes were found. EMR, electronic medical record; Jr KwikPen, Junior KwikPen; T1D, Type 1 diabetes; T2D, Type 2 diabetes.

At the time of the index date, IQVIA's EMRs database included a total of 1,190,000 individuals, of whom 107,370 had diabetes (7,220 T1D; 99,982 T2D; 168 gestational diabetes; Figure 2). A total of 416 patients (326 T1D and 90 T2D) initiated treatment with Jr KwikPen within the inclusion period, which extended from 1 May 2018, (launch date in Spain) to 31 December 2020. Within this period, none of the patients who initiated treatment with Jr KwikPen were identified as having gestational diabetes (Figure 2). Six patients with T1D and four patients with T2D who initiated treatment with Jr KwikPen were pregnant (Table 1). These were patients who had T1D or T2D already and then became pregnant, not patients who developed gestational diabetes during a pregnancy. No patients were identified as frail, but 20 (80%) patients with T1D and 23 (88.5%) patients with T2D in the ≥65‐year age range were identified as having pre‐frailty. All 416 patients included in the study initiated Jr KwikPen between May 2018 (launch date in Spain) and December 2020. The proportion of patients among the overall sample who initiated treatment with Jr KwikPen each year increased from 16.6% in 2018 to 46.4% in 2019, with a decrease in 2020 (37.0%) coinciding with the coronavirus disease‐2019 (COVID‐19) pandemic (Table 2).

**TABLE 1:** Sociodemographic and clinical characteristics of patients who initiated treatment with Jr KwikPen by type of diabetes and age range.

Variable	T1D				T2D			Total T1D and T2D
	Age range in years				Age range in years			
	<18	18–64	≥65	Total	18–64	≥65	Total	
	*N* = 107	*N* = 194	*N* = 25	*N* = 326	*N* = 64	*N* = 26	*N* = 90	*N* = 416
Age (years)	8.9 (4.2)	40.4 (11.8)	71.2 (5.7)	32.5 (20.7)	47.4 (11.5)	75.5 (7.6)	55.5 (16.6)	37.4 (22.0)
Gender (male), *N* (%)	53 (49.5)	67 (34.5)	5 (20.0)	125 (38.3)	20 (31.3)	11 (42.3)	31 (34.4)	156 (37.5)
Gender (female), *N* (%)	54 (50.5)	127 (65.5)	20 (80.0)	201 (61.7)	44 (68.8)	15 (57.7)	59 (65.6)	260 (62.5)
Time since first diabetes record^a^ (years)	1.0 (2.1)	8.1 (4.4)	8.6 (4.5)	5.8 (5.1)	7.8 (4.1)	8.3 (4.4)	7.9 (4.2)	6.3 (5.0)
Newly diagnosed,^b^ *N* (%)	57 (53.3)	8 (4.1)	4 (16.0)	69 (21.2)	–	2 (7.7)	2 (2.2)	71 (17.1)
Weight (kg)	30.6 (14.4)	61.7 (11.7)	68.6 (13.2)	50.1 (20.3)	69.2 (13.9)	65.6 (18.7)	68.1 (15.4)	54.5 (20.7)
BMI Valid *N* (%)	92 (86.0)	128 (66.0)	19 (76.0)	239 (73.3)	54 (84.4)	23 (88.5)	77 (85.6)	316 (76.0)
BMI (kg/m^2^)	17.3 (2.4)	22.8 (3.0)	26.1 (3.8)	20.9 (4.2)	25.1 (4.2)	25.4 (5.5)	25.2 (4.6)	22.0 (4.6)
BMI category,^c^ *N* (%)								
Underweight	–	7 (5.5)	–	7 (4.8)	3 (5.6)	1 (4.3)	4 (5.2)	11 (4.9)
Normal weight	–	92 (71.9)	7 (36.8)	99 (67.3)	21 (38.9)	8 (34.8)	29 (37.7)	128 (57.1)
Overweight	–	26 (20.3)	9 (47.4)	35 (23.8)	26 (48.1)	10 (43.5)	36 (46.8)	71 (31.7)
Obese	–	3 (2.3)	3 (15.8)	6 (4.1)	4 (7.4)	4 (17.4)	8 (10.4)	14 (6.3)
Class I	–	3 (2.3)	2 (10.5)	5 (3.4)	3 (5.6)	3 (13.0)	6 (7.8)	11 (4.9)
Class II	–	–	1 (5.3)	1 (0.7)	1 (1.9)	1 (4.3)	2 (2.6)	3 (1.3)
HbA1c Valid *N* (%)	9 (8.4)	119 (61.3)	13 (52.0)	141 (43.3)	44 (68.8)	20 (76.9)	64 (71.1)	205 (49.3)
HbA1c (%)	9.5 (3.9)	7.7 (1.5)	7.6 (1.1)	7.8 (1.7)	7.9 (1.5)	8.3 (1.5)	8.0 (1.5)	7.9 (1.7)
HbA1c <7.0%, *N* (%)	3 (33.3)	43 (36.1)	5 (38.5)	51 (36.2)	12 (27.3)	4 (20.0)	16 (25.0)	67 (32.7)
HbA1c ≥7.0%, *N* (%)	6 (66.7)	76 (63.9)	8 (61.5)	90 (63.8)	32 (72.7)	16 (80.0)	48 (75.0)	138 (67.3)
Total cholesterol (mg/day)^d^	168.00 (31.25)	192.58 (45.20)	199.17 (35.71)	190.33 (43.57)	183.92 (37.71)	163.28 (31.75)	177.29 (36.91)	186.38 (42.00)
HDL cholesterol (mg/dL)^d^	100.42 (48.46)	84.57 (35.28)	91.58 (52.51)	86.77 (38.48)	85.19 (45.39)	82.11 (40.17)	84.17 (43.36)	85.98 (39.91)
LDL cholesterol (mg/dL)^d^	104.30 (25.63)	111.59 (35.99)	118.00 (36.63)	111.57 (35.15)	107.20 (32.39)	85.39 (28.07)	99.79 (32.44)	107.88 (34.66)
Triglycerides (mg/dL)^d^	107.64 (100.80)	78.25 (46.13)	95.33 (42.01)	83.10 (54.69)	93.46 (49.39)	146.83 (117.77)	110.93 (81.44)	91.51 (65.00)
GFR (mL/min/1.73 m^2^)^d^	149.40 (24.45)	98.08 (16.96)	66.36 (20.02)	102.50 (28.00)	93.90 (19.84)	71.53 (21.69)	86.21 (22.97)	97.74 (27.59)
Presence of diabetes associated comorbidities, *N* (%)	5 (4.7)	65 (33.5)	21 (84.0)	91 (27.9)	36 (56.3)	22 (84.6)	58 (64.4)	149 (35.8)
Abnormal blood chemistry	4 (3.7)	16 (8.2)	4 (16.0)	24 (7.4)	8 (12.5)	4 (15.4)	12 (13.3)	36 (8.7)
Acute coronary syndrome	–	3 (1.5)	4 (16.0)	7 (2.1)	4 (6.3)	5 (19.2)	9 (10.0)	16 (3.8)
Cardiac ischaemia	–	3 (1.5)	4 (16.0)	7 (2.1)	4 (6.3)	5 (19.2)	9 (10.0)	16 (3.8)
Carotid arterial disease	–	1 (0.5)	–	1 (0.3)	–	–	–	1 (0.2)
Chronic kidney disease stages	–	1 (0.5)	1 (4.0)	2 (0.6)	–	2 (7.7)	2 (2.2)	4 (1.0)
Congestive heart failure	–	1 (0.5)	–	1 (0.3)	1 (1.6)	4 (15.4)	5 (5.6)	6 (1.4)
Hyperlipidemia	‐	38 (19.6)	11 (44.0)	49 (15.0)	23 (35.9)	15 (57.7)	38 (42.2)	87 (20.9)
Hypertension	1 (0.9)	22 (11.3)	15 (60.0)	38 (11.7)	13 (20.3)	19 (73.1)	32 (35.6)	70 (16.8)
Ischaemic stroke	–	1 (0.5)	–	1 (0.3)	1 (1.6)	3 (11.5)	4 (4.4)	5 (1.2)
Left ventricular dysfunction	–	–	–	–	–	2 (7.7%)	2 (2.2%)	2 (0.5%)
Microalbuminuria	1 (0.9)	3 (1.5)	1 (4.0)	5 (1.5)	–	–	–	5 (1.2)
Myocardial infarction	–	1 (0.5)	–	1 (0.3)	2 (3.1)	1 (3.8)	3 (3.3)	4 (1.0)
Peripheral artery disease	–	7 (3.6)	1 (4.0)	8 (2.5)	–	2 (7.7)	2 (2.2)	10 (2.4)
Retinopathy	–	5 (2.6)	1 (4.0)	6 (1.8)	1 (1.6)	–	1 (1.1)	7 (1.7)
Transient ischaemic attack	–	–	–	–	–	3 (11.5)	3 (3.3)	3 (0.7)
Unstable angina	–	–	1 (4.0)	1 (0.3)	–	1 (3.8)	1 (1.1)	2 (0.5)
Pregnant, *N* (%)	–	6 (4.7)	–	6 (3.0)	4 (9.1)	–	4 (6.8)	10 (3.8)
Frail patients^e^								
Non‐frailty	–	119 (61.3)	5 (20.0)	124 (56.6)	32 (50.0)	3 (11.5)	35 (38.9)	159 (51.5)
Pre‐frailty	‐	75 (38.7)	20 (80.0)	95 (43.4)	32 (50.0)	23 (88.5)	55 (61.1)	150 (48.5)

*Note*: Data are mean (SD) unless otherwise stated.

Abbreviations: BMI, body mass index; GFR, glomerular filtration rate; HbA1c, glycosylated haemoglobin; HDL, high‐density lipoprotein; Jr KwikPen, Junior KwikPen; LDL, low‐density lipoprotein; SD, standard deviation; T1D, Type 1 diabetes; T2D, Type 2 diabetes.

^a^ First register of diabetes diagnosis in 2008 was considered the date of diabetes diagnosis but diagnosis could be before 2008.

^b^ Patients meeting the following criteria were considered newly diagnosed: the date of diagnosis coincided with the index date, no record of diabetes or antidiabetic treatment prior to the index date, and antidiabetic treatment records within the 15 days before the index date but with no record of antidiabetic prescription before the 15‐day time window.

^c^ Underweight (BMI is less than 18.5 kg/m^2^), Normal weight (BMI is 18.5–24.9 kg/m^2^), Overweight (BMI is 25–29.9 kg/m^2^), Obese (BMI is 30 kg/m^2^ or more), Class I Obese (BMI is 30–34.9 kg/m^2^), Class II Obese (BMI is 35–39.9 kg/m^2^), Class III Obese (BMI is 40 kg/m^2^ or higher). BMI is not available for patients <18 years old.

^d^ Most recent values in the database before index date were used.

^e^ Evaluation of frailty was performed with the FRAIL scale: Non‐frailty (0 components), Pre‐frailty (1–2 components), Frailty (>2 components). No patients were identified as having ‘frailty’. ‘Frailty’ is not estimated for patients <18 years old.

**TABLE 2:** Treatment initiation characteristics.

Variable	T1D				T2D			Total T1D and T2D
	Age range in years				Age range in years			
	<18	18–64	≥65	Total	18–64	≥65	Total	
	*N* = 107	*N* = 194	*N* = 25	*N* = 326	*N* = 64	*N* = 26	*N* = 90	
								*N* = 416
Year of Jr KwikPen treatment initiation								
2018	13 (12.1)	36 (18.6)	6 (24.0)	55 (16.9)	8 (12.5)	6 (23.1)	14 (15.6)	69 (16.6)
2019	52 (48.6)	91 (46.9)	10 (40.0)	153 (46.9)	29 (45.3)	11 (42.3)	40 (44.4)	193 (46.4)
2020	42 (39.3)	67 (34.5)	9 (36.0)	118 (36.2)	27 (42.2)	9 (34.6)	36 (40.0)	154 (37.0)
Prescriber								
Emergency	–	1 (0.5)	–	1 (0.3)	–	–	–	1 (0.2)
Endocrinologist	7 (6.5)	160 (82.5)	16 (64.0)	183 (56.1)	48 (75.0)	13 (50.0)	61 (67.8)	244 (58.7)
Nephrologist	–	–	1 (4.0)	1 (0.3)	–	–	–	1 (0.2)
Oncologist	–	–	1 (4.0)	1 (0.3)	–	–	–	1 (0.2)
Pediatrician^a^	94 (87.9)	–	–	94 (28.8)	–	–	–	94 (22.6)
Primary care	6 (5.6)	33 (17.0)	7 (28.0)	46 (14.1)	16 (25.0)	13 (50.0)	29 (32.2)	75 (18.0)
Daily dose of Jr KwikPen at initiation (UI/day)	25.61 (18.73)	27.94 (15.95)	20.76 (7.94)	26.63 (16.56)	25.00 (14.96)	16.62 (6.42)	22.58 (13.59)	25.75 (16.03)
Daily dose of Jr KwikPen at initiation (UI/day) categories								
<15 UI/day	21 (19.6)	20 (10.3)	5 (20.0)	46 (14.1)	14 (21.9)	9 (34.6)	23 (25.6)	69 (16.6)
15− < 30 UI/day	52 (48.6)	96 (49.5)	14 (56.0)	162 (49.7)	26 (40.6)	16 (61.5)	42 (46.7)	204 (49.0)
30− < 45 UI/day	20 (18.7)	53 (27.3)	6 (24.0)	79 (24.2)	18 (28.1)	1 (3.8)	19 (21.1)	98 (23.6)
45+ UI/day	14 (13.1)	25 (12.9)	–	39 (12.0)	6 (9.4)	–	6 (6.7)	45 (10.8)
Daily dose of Jr KwikPen, UI/kg (SD)	1.04 (1.13)	0.45 (0.27)	0.31 (0.13)	0.67 (0.79)	0.39 (0.24)	0.28 (0.12)	0.35 (0.22)	0.59 (0.71)
Injections per day, mean (SD)	2.97 (0.50)	2.85 (0.58)	2.88 (0.33)	2.89 (0.54)	2.84 (0.54)	3.00 (0.00)	2.89 (0.46)	2.89 (0.52)
Injections per day, median	3.0	3.0	3.0	3.0	3.0	3.0	3.0	3.0

*Note*: Data are *N* (%) unless otherwise stated.

Abbreviations: Jr KwikPen, Junior KwikPen; SD, standard deviation; T1D, Type 1 diabetes; T2D, Type 2 diabetes.

^a^ This category includes primary care providers and endopediatricians.

For patients with T1D (*N* = 326), the mean age was 32.5 years, 59.5% were aged 18–64 years, 32.8% were below 18 years old (Table 1), and 61.7% were female. Of the 326 patients with T1D in the study, 69 (21.2%) were newly diagnosed at Jr KwikPen treatment initiation. Time from first diabetes record in the database was 10.0–19.9 years in 36.8% and 5.0–9.9 years in 14.7% of patients. At Jr KwikPen treatment initiation, the mean number of Jr KwikPen injections per day was 2.89, and the daily dose was 26.63 UI/day or 0.67 UI/kg (Table 2).

For patients with T2D (*N* = 90), the mean age was 55.5 years, 71.1% were aged 18–64 years, 28.9% were ≥ 65 years old (Table 1), and 65.6% were female. Only two patients newly initiated treatment with Jr KwikPen at the time of T2D diagnosis. Time from first diabetes record in the database was 10.0–19.9 years in 53.3% and 1.0–4.9 years in 24.4% of patients with T2D. For those ≥65 years old, the time from first diabetes record was 8.3 years. At Jr KwikPen treatment initiation, the mean number of Jr KwikPen injections per day was 2.89, and the daily dose was 22.58 UI/day or 0.35 UI/kg (Table 2).

The mean HbA1c value was 7.8% for patients with T1D and 8.0% for those with T2D (Table 1). Comorbidities were present in 27.9% of patients with T1D and 64.4% of those with T2D (Table 1). The most prevalent comorbidities in both diabetes groups were hyperlipidemia (20.9%) and hypertension (16.8%). The frequency of comorbidities was generally greater in the older age groups.

### 3.2 Adults using Jr KwikPen

**FIGURE 3:**
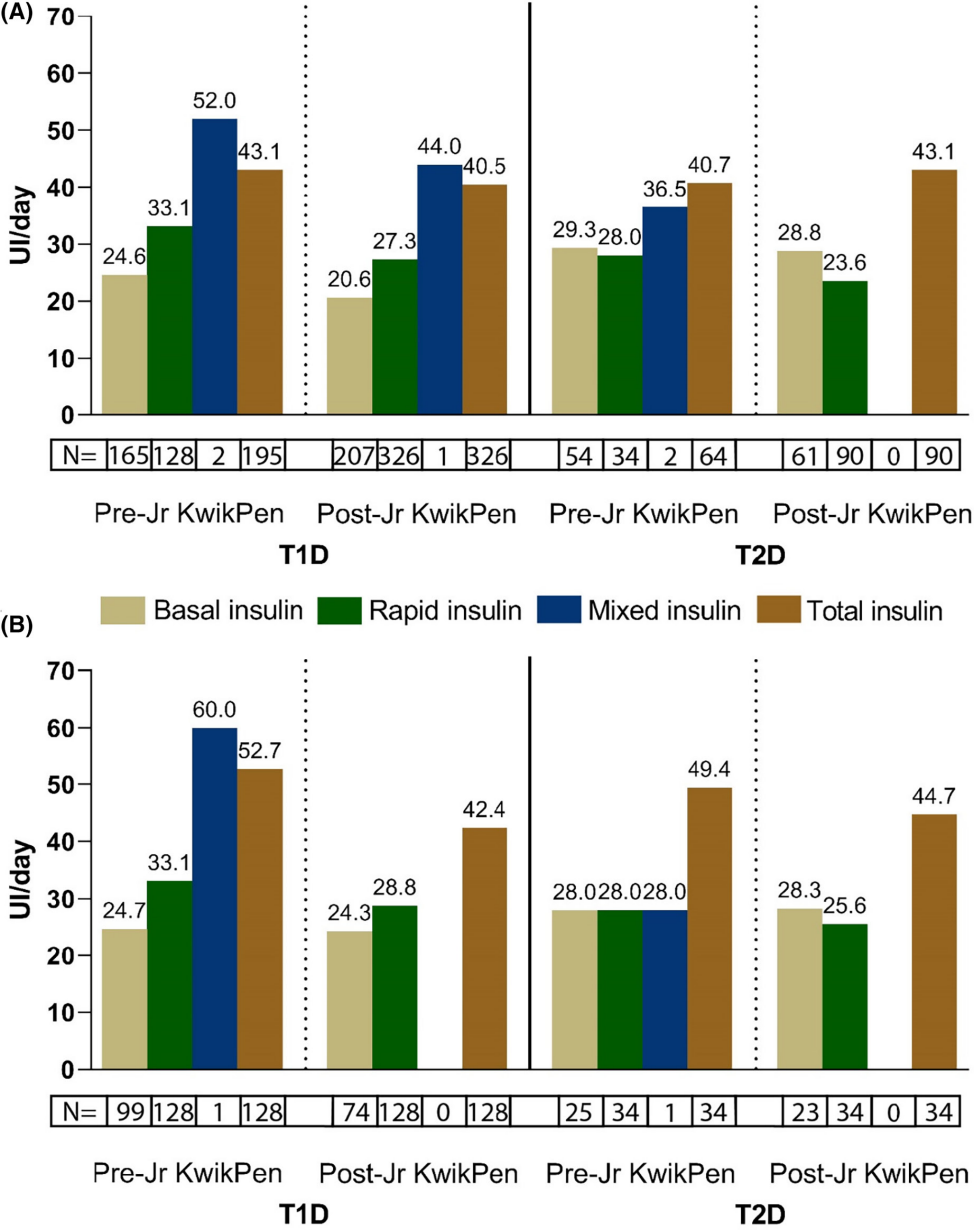
A. Mean daily dose of insulin prescribed within the 90 days before and within the 60 days after Jr KwikPen treatment initiation in newly and previously diagnosed patients with T1D and T2D. B. Mean daily dose of insulin prescribed within the 90 days before and within the 60 days after Jr KwikPen treatment initiation in previously diagnosed patients with T1D and T2D treated with rapid insulin prior to Jr KwikPen initiation. Data are mean (SD). Jr KwikPen, Junior KwikPen; SD, standard deviation; T1D, Type 1 diabetes; T2D, Type 2 diabetes.

For 326 patients with T1D, endocrinologists were the most frequent prescribers (82.5% for those aged 18–64 years and 64% for those aged ≥65 years; Table 2). Previous insulin was prescribed in 178 (95.7%) patients aged 18–64 years and 21 (100%) patients aged ≥65 years (Figure S1, Table S2). The percentage of patients aged18‐64 years and ≥ 65 years receiving different types of glucose‐lowering/antihyperglycemic prescriptions within 60 days of Jr KwikPen treatment initiation was similar to that in the overall T1D population (Figure S2, Table S3). The mean daily dose of total insulin after Jr KwikPen treatment initiation was similar to that before Jr KwikPen treatment initiation overall and within age groups (Figure 3, Table S4). At Jr KwikPen treatment initiation, the mean number of Jr KwikPen injections per day and daily dose were 2.85 and 27.94 UI/day or 0.45 UI/kg, respectively, for those aged 18–64 years and 2.88 and 20.76 UI/day or 0.31 UI/kg, respectively, for those aged ≥65 years (Table 2).

Patients with T2D included in the study (*N* = 90) who initiated treatment with Jr KwikPen each year increased from 15.6% in 2018 to 44.4% in 2019, with a decrease in 2020 (40.0%) coinciding with the COVID‐19 pandemic (Table 2). For patients with T2D (*N* = 90), 67.8% of prescribers were endocrinologists, followed by general practitioners (GPs) who prescribed Jr KwikPen in 32.2% of patients (Table 2). GPs were more represented as prescribers in the older age groups (28.0% in T1D and 50.0% in T2D). Previous insulin was prescribed in 81 (92.0%) patients with T2D (Figure S1, Table S2). The mean daily dose of total insulin after Jr KwikPen treatment initiation was similar to that before Jr KwikPen treatment initiation overall and within age groups (Figure 3, Table S4). At Jr KwikPen treatment initiation, the mean number of Jr KwikPen injections per day and daily dose were 2.84 and 25.00 UI/day or 0.39 UI/kg, respectively, for those aged 18–64 years and 3 and 16.62 UI/day or 0.28 UI/kg, respectively, for those aged ≥65 years (Table 2).

### 3.3 Paediatric patients (with T1D <18 years old) using Jr KwikPen

From 326 patients with T1D, 69 were considered newly diagnosed and 57 were < 18 years old (Table 1). The proportion of patients with T1D aged <18 years included in the study who initiated treatment with Jr KwikPen each year increased from 12.1% in 2018 to 48.6% in 2019, with a decrease in 2020 (39.3%) coinciding with the COVID‐19 pandemic (Table 2). The main prescribers were paediatricians (87.9%; Table 2). Previous insulin was prescribed in 46 (92.0%) patients (Figure S1, Table S2). The mean daily dose of total insulin after treatment initiation with Jr KwikPen was similar to that before Jr KwikPen use (Table S4). At treatment initiation with Jr KwikPen, the mean number of Jr KwikPen injections per day was 2.97 and the daily dose was 25.61 UI/day or 1.04 UI/kg (Table 2).

## 4 DISCUSSION

This is the first real‐world study to describe the profile of patients with T1D and T2D using Jr KwikPen in Europe. Jr KwikPen users were mostly children or adults aged 18–65 years, with T1D, female and had poor glycemic control at treatment initiation. Diabetes‐associated comorbidities were present in about a third of the population overall, but in 84%–85% of the users greater than 65 years old. These results suggest that Humalog Jr KwikPen is prescribed for use in adults, adolescents, and children with T1D or T2D who may benefit from finer insulin dose adjustments.

The proportion of patients in the total study sample who initiated treatment with Jr KwikPen increased from 2018 to 2019 and decreased in 2020, which coincided with the COVID‐19 pandemic. About 5% of patients with T1D in IQVIA's database, initiated treatment with Jr KwikPen in the study period. A third of the identified Jr KwikPen users with T1D were children and adolescents. Jr KwikPen users were younger than the T1D population included in the study by Piras de Oliveira et al.^31^ and the T2D population in Spain.^32^, ^33^, ^34^ Piras de Oliveira et al. conducted a survey of adults with T1D and caregivers of minors with T1D in the United States to understand the user and their perspectives on half‐unit pens.^31^ Jr KwikPen was most commonly prescribed by paediatricians for paediatric patients (87.9% for patients with T1D <18 years old) and by endocrinologists for adult patients (82.5% for patients with T1D 18–64 years old, 64.0% for patients with T1D ≥65 years old, and 67.8% for patients with T2D; Table 2).

Comorbidities were present in 27.9% of patients with T1D and 64.4% of those with T2D, with hyperlipidemia and hypertension being the most frequent in both groups. In a study of patients with T1D in Spain 48.7% overall had comorbidities or complications associated with T1D and 52.9% of patients greater than 18 years old had comorbidities or complications.^35^ The most common complications were retinopathy, hypothyroidism and dyslipidemia. In a study of patients with T2D in Catalonia 71.9% had hypertension and 61.1% had hyperlipidemia.^34^ Mean glycemic control was above the standard target of less than 7.0% in both patients with T1D and T2D at Jr KwikPen treatment initiation (7.8% and 8.0%, respectively), with 63.8% and 75.0% of patients with T1D and T2D having glycemic control above the target. In the SED1 study of patients with T1D in Spain mean HbA1c was 7.6% with 29.7% of patients with HbA1c <7%.^35^ In the Catalonian T2D study mean HbA1c was 7.1%, 34.7% of patients had HbA1c <6.5% and 20.7% of patients had HbA1c 6.5%–6.9%.^34^ In our study a total of 4.1% of patients with T1D and 10.4% of patients with T2D had a BMI ≥30 kg/m^2^, which was substantially below that of almost half the population reported by Represas Carrera et al. for T2D.^32^ Represas Carrera et al. described the sociodemographic and clinical characteristics, metabolic control of the disease, and comorbidity and cardiovascular risk of patients with T2D in Vigo, Spain. However, HbA1c and BMI data should be cautiously interpreted because of the amount of missing data.

A recent study demonstrated the benefits of half‐unit pens in preventing hypoglycemic and hyperglycemic events as well as the user's perception and showed a decrease in worry and anxiety.^31^ The use of disposable pens has increased over time.^36^ Insulin pens have been associated with increased adherence and persistence, which are in turn associated with lower HbA1c values and fewer hypoglycemic events than those with other administered forms of insulin. Pens have also been associated with economic benefits compared to other administered forms of insulin. Several smartphone apps have been launched to facilitate a more exact bolus dose calculation for people utilizing insulin and which may be more effectively delivered using pen devices capable of delivering precise half‐unit increments.^20^ However, there is little evidence on the use of Jr KwikPen under routine care conditions in the post‐marketing setting.

The main limitations of this study derive from the retrospective nature of the design and the use of an existing EMR database as the data source, with missing data, heterogeneity in terms of data quality, variability in the frequency of data capture and coverage of key study‐related parameters. Physicians contributing to panels who are included in the database may not be fully representative of the physician population in Spain, as data are only collected from those who decide to participate in the panel. These physicians (and subsequently their patients) may differ from those who do not contribute in ways that are unmeasured, resulting in potential selection bias. The high proportion of paediatricians and GPs prescribing Jr KwikPen in the study may be related to their high representation in the database as well as to the potential for induced prescription from endocrinologists. This may present challenges in identifying patients with diabetes and classifying them as having T1D or T2D within the data set. Given the nature of real‐world data and variabilities in efficiency and completeness of records, missing data are likely to be present. Additionally, as concomitant prescriptions were obtained specifically from the 60‐day post‐index period, some medications may have fallen out of this time window, resulting in the underrepresentation of some treatments (e.g. basal insulin) in the concomitant analysis. Furthermore, the mean daily dose of insulin prescribed may differ from the amount of insulin that patients actually use or receive.^37^ Another limitation of the study was the number of missing values for HbA1c and BMI at Jr KwikPen treatment initiation (Table 1). For 53% of paediatric patients, HbA1c at initiation was also HbA1c at diagnosis. BMI category was not included for paediatric patients.

## 5 CONCLUSION

This is the first study to describe the profile of patients treated with insulin lispro 100 units/mL Jr KwikPen in clinical practice in Europe. The profile of patients who initiated therapy with Jr KwikPen in routine practice was broad with many patients being adults aged 18–65 years. Most of these patients had T1D, inadequate glycemic control, and multiple associated comorbidities. Gestational diabetes was not found in any patients, but 10 Jr KwikPen users were pregnant. Jr KwikPen was first prescribed mainly by paediatricians and endocrinologists or GPs for those older than 18 years.

These real‐world results suggest that Humalog Jr KwikPen is prescribed in patients who may benefit from finer insulin dose adjustments, including children, adolescents, elderly people and pregnant women with T1D or T2D.

## AUTHOR CONTRIBUTIONS

**F. Javier Ampudia‐Blasco:** Writing – original draft (equal); writing – review and editing (equal). **Natalia Duque:** Conceptualization (equal); methodology (equal); writing – original draft (equal); writing – review and editing (equal). **Esther Artime:** Conceptualization (equal); methodology (equal); writing – original draft (equal); writing – review and editing (equal). **Elena Caveda:** Conceptualization (equal); methodology (equal); writing – original draft (equal); writing – review and editing (equal). **Erik Spaepen:** Conceptualization (equal); methodology (equal); writing – original draft (equal); writing – review and editing (equal). **Silvia Díaz‐Cerezo:** Conceptualization (equal); methodology (equal); writing – original draft (equal); writing – review and editing (equal). **Miriam Rubio‐de Santos:** Conceptualization (equal); methodology (equal); writing – original draft (equal); writing – review and editing (equal). **Daniel Callejo Velasco:** Formal analysis (equal); writing – original draft (equal); writing – review and editing (equal). **M. Pilar Bahíllo Curieses:** Writing – original draft (equal); writing – review and editing (equal).

## FUNDING INFORMATION

This study was supported by Eli Lilly and Company.

## CONFLICT OF INTEREST STATEMENT

Natalia Duque, Esther Artime, Elena Caveda, Silvia Díaz, and Miriam Rubio are employees and shareholders of Eli Lilly and Company. Erik Spaepen is a consultant to Eli Lilly and Company. F. Javier Ampudia‐Blasco has served on advisory panels for Abbott, AstraZeneca, Boehringer Ingelheim, Eli Lilly and Company, GlaxoSmithKline, LifeScan, Medtronic, Merck, Novartis, Novo Nordisk, Pfizer, Roche, and Sanofi and has received research support from Abbott, AstraZeneca, Boehringer Ingelheim, Bayer, Eli Lilly and Company, GlaxoSmithKline, LifeScan, Merck, Novo Nordisk, Pfizer, Sanofi, and Servier. Daniel Callejo is an employee of IQVIA. IQVIA has received funding from Eli Lilly and Company to conduct the study reported in the manuscript. M Pilar Bahíllo‐Curieses has received honoraria as a speaker from Eli Lilly and Company, Novo Nordisk, Medtronic, Roche, Abbot, and Sandoz.

## ETHICS STATEMENT

The study was approved by the accredited Clinical Research Ethics Committees of the Hospital Clínic de Barcelona before study initiation. The study was also conducted according to Good Clinical Practice guidelines (International Conference of Harmonization) and the Declaration of Helsinki.

## Supporting information


Data S1.

## Data Availability

The datasets generated during and/or analysed during the current study are not publicly available, as this is a commercially available database, and is not directly accessible by third parties.
