# Supplementary material for: Which people with diabetes are treated with a disposable, half‐unit insulin pen? A real‐world, retrospective, database study in Spain
Source: Endocrinol Diabetes Metab. 2023 Sep 15;6(6):e451. doi: 10.1002/edm2.451 (PMC10638621; doi:10.1002/edm2.451)
Supplement: Supplementary file 1 — Data S1. [file EDM2-6-e451-s001.docx]

**Which people with diabetes are treated with a disposable, half-unit insulin pen? A real-world, retrospective, database study in Spain**

**Supplementary Materials**

F. Javier Ampudia-Blasco^1^, Natalia Duque^2^, Esther Artime^2^, Elena Caveda^2^, Erik Spaepen^3^, Silvia Díaz-Cerezo^2^, Miriam Rubio-de Santos^2^, Daniel Callejo Velasco^4^, M Pilar Bahíllo-Curieses^5^

^1^Endocrinology and Nutrition Department, Clinic University Hospital Valencia, INCLIVA Research Foundation, Valencia, Spain

^2^Eli Lilly and Company, Alcobendas, Madrid, Spain

^3^HaaPACS GmbH, Schriesheim, Germany

^4^IQVIA, Madrid, Spain

^5^Servicio de Pediatría, Endocrinología Pediátrica, Hospital Clínico Universitario de Valladolid, Valladolid, Spain

Corresponding Author: Natalia Duque

Address: Eli Lilly and Company

Alcobendas

Madrid

Spain

Phone: +34 916 63 34 67

E-mail: duque_natalia@lilly.com

**Supplementary Methods**

***Study Design and Population***

Data were extracted for the pre-index period (medical history data and antidiabetic prescriptions prior to the start of treatment with Junior KwikPen [Jr KwikPen], which extended from diabetes diagnosis to last data available prior to the index date) and index date (patient’s sociodemographic and clinical characteristics, status at the start of treatment with Jr KwikPen, and concomitant antidiabetic prescriptions). For each patient, a verification period of +2 months from the index date was considered to allow for the verification of concomitant prescriptions and other descriptive variables such as pregnancy. Data up to February 28, 2021, were extracted to allow for a +2-month verification period for all patients in the study.

***Variables***

Patients’ sociodemographic and clinical characteristics were derived from available data at the index date or from the last data available before the index date. Patients’ sociodemographic characteristics at index included age, sex, and smoking status.

Clinical status at index included body mass index and weight; laboratory values (glycosylated hemoglobin, estimated glomerular filtration rate, glycemia, low-density and high-density cholesterol, and triglycerides); diabetes-related comorbidities (hypertension, hyperlipidemia, acute coronary syndrome, cardiac ischemia, myocardial infarction, peripheral artery disease, abnormal blood chemistry, chronic kidney disease stages 3-5, unstable angina, congestive heart failure, transient ischemic attack, ischemic stroke, microalbuminuria, retinopathy, carotid arterial disease, left ventricular dysfunction, left ventricular hypertrophy, others); frailty-related diagnoses (fatigue [including neurasthenia, malaise, senility w/o psychosis, other general symptoms]; resistance [unspecified debility]; ambulation [difficulty walking, abnormal gait]; illnesses [hypertension, cancer, chronic obstructive pulmonary disease, acute myocardial infarction, chronic heart failure, angina, asthma, arthritis, stroke, chronic kidney disease]; body weight loss [Kwashiorkor, nutritional marasmus, other severe protein-calorie malnutrition, malnutrition of moderate and mild degree, unspecified protein-calorie malnutrition, muscular wasting and disuse atrophy not elsewhere classified, early satiety, loss of weight, feeding difficulties and mismanagement, adult failure to thrive, cachexia]); pregnancy status (identification through ICD-9-CM codes) (Table S1) during treatment with Jr KwikPen - Yes/No.

Prior medical and treatment history (pre-index period) included diabetes diagnosis (type 1 diabetes [T1D] or type 2 diabetes [T2D] diagnosis date or first diabetes record available in the database. Time between the first diagnosis of T1D/T2D [or first diabetes record in the database] and the index date was calculated); prior insulin prescriptions available in the database between diabetes diagnosis (or first diabetes record) and the index date - Yes/No (% naive patients at the index date). If Yes, insulin prescriptions within 90 days prior to the index date: insulin type and doses - rapid insulin (NovoRapid®, Fiasp®, Apidra®, Humalog® vial, Humalog 100, Humalog 200, Humulina, Actrapid), basal insulin (Abasaglar, Humulina® NPH, Insulatard NPH, Lantus®, Levemir, Toujeo®, Tresiba®, Semglee®), mixed insulin (Humalog Mix, Humulina 30/70, Mixtard, NovoMix®); prior non-insulin antidiabetic prescriptions available in the database between diabetes diagnosis (or first diabetes record) and the index date - Yes/No. If Yes: non-insulin antidiabetic prescriptions within 90 days prior to the index date type and specific drug (if applicable) - metformin, sulfonylureas, sodium-glucose transport protein 2 (SGLT2) inhibitors, dipeptidyl peptidase 4 (DPP-4) inhibitors, glucagon-like peptide 1 receptor agonists (GLP-1 RAs), prandial glucose regulators, alpha glucosidase inhibitors, and glitazone.

Humalog Junior KwikPen® treatment (index date) included Jr KwikPen index date, daily dose (UI/day and UI/kg/day), and frequency of administration; concomitant insulin prescriptions. Insulin regimen at the index date or available prescriptions recorded within the 60 days after the index date - Yes/No. If Yes, all available insulin prescriptions recorded within the 60 days after the index date were collected as types and doses - rapid insulin (NovoRapid, Fiasp, Apidra, Humalog vial, Humalog 100, Humalog 200, Humulina, Actrapid), basal insulin (Abasaglar, Humulina NPH, Insulatard NPH, Lantus, Levemir, Toujeo, Tresiba, Semglee), mixed insulin (Humalog Mix, Humulina 30/70, Mixtard, NovoMix); concomitant non-insulin antidiabetic prescriptions. Non-insulin antidiabetic prescriptions at the index date or available prescriptions recorded within the 60 days after the index date - Yes/No. If Yes, all available non-insulin antidiabetic prescriptions recorded within the 60 days after the index date will be collected - type and specific drug and start date (if applicable), metformin, SGLT2 inhibitors, DPP-4 inhibitors, GLP-1 RAs, prandial glucose regulators, alpha glucosidase inhibitors, and glitazone.

Jr KwikPen Prescription Initiator (index date) included general practitioners/specialists who initiated the prescription of Jr KwikPen at the index date.

***Database***

IQVIA’s EMR database only contains data from 2008 onwards, which limited the availability of data from the past (diagnosis date, prior treatments, etc.). Therefore, certain assumptions had to be made for the purpose of the analysis, for example, the date of diagnosis for patients diagnosed before 2008 was imputed to the date of the first record available in IQVIA’s EMR database and is referred to as the time from first diabetes record.


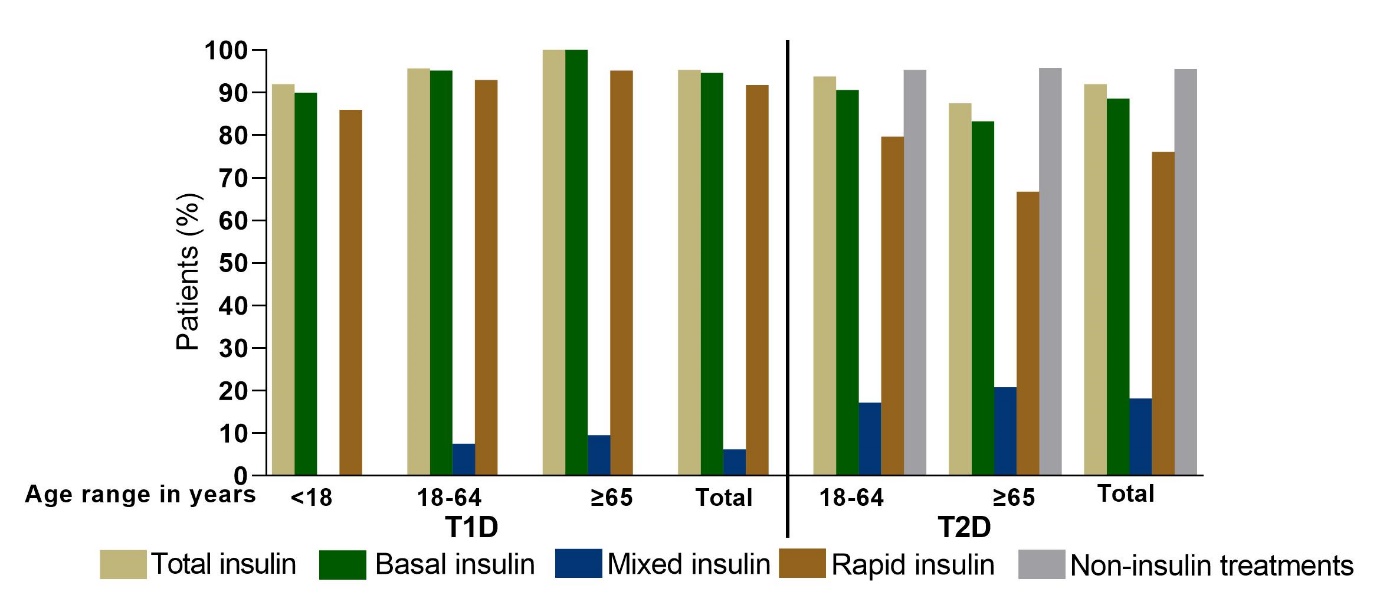
 Figure S1. Percentage of patients receiving different types of glucose-lowering/antihyperglycemic prescriptions before initiation of treatment with Jr KwikPen by age range and type of diabetes. Jr KwikPen, Junior KwikPen; T1D, type 1 diabetes; T2D, type 2 diabetes.


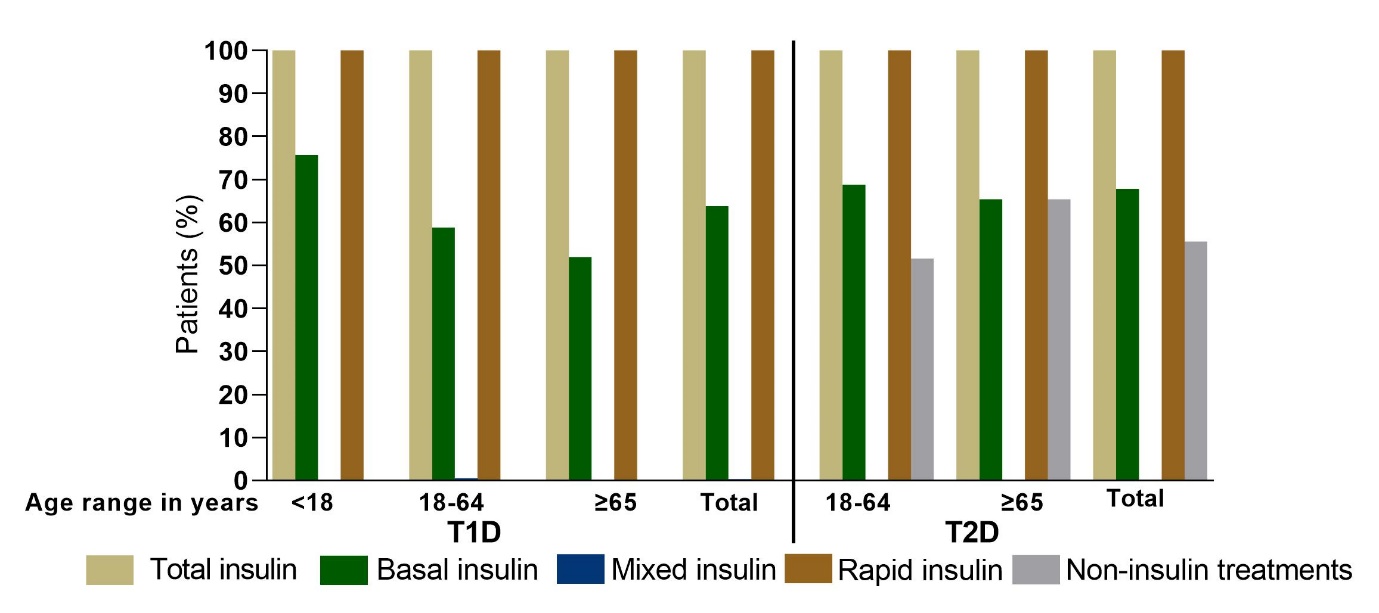


Figure S2. Percentage of patients receiving different types of glucose-lowering/antihyperglycemic prescriptions, including Jr KwikPen, within the 60 days from Jr KwikPen treatment initiation by age range and type of diabetes. Jr KwikPen, Junior KwikPen; T1D, type 1 diabetes; T2D, type 2 diabetes.

| **Table S1. Diagnostic codes to identify variables** | |
| --- | --- |
| **Description** | **ICD9-CM codes** |
| **Diagnostic codes used to identify diabetes mellitus** | |
| Diabetes mellitus, type 1 not stated as controlled | 250.x1 |
| Diabetes mellitus, type 1, uncontrolled | 250.x3 |
| Diabetes mellitus, type 2 not stated as controlled | 250.x0 |
| Diabetes mellitus, type 2, uncontrolled | 250.x2 |
| Abnormal glucose tolerance of mother complicating pregnancy childbirth or the puerperium | 648.8 |
| **Diagnostic codes used to identify pregnancy** | |
| Complications of pregnancy, childbirth, and the puerperium | 630.xx-679.xx |
| Normal pregnancy | V22.xx |
| Supervision of high-risk pregnancy | V23.xx |
| **Diagnostic codes used to identify frailty** | |
| Fatigue | 300.5, 780.7x, 797, 780.99 |
| Resistance | 799.3, |
| Ambulation | 719.7, 781.2 |
| Illnesses | Hypertension (401.x–405.x);  Cancer (140.x–208.x);  COPD (491.x);  Acute myocardial infarction (410.x);  Congestive heart failure (428.x);  Angina (411.1, 411.81, 411.89, 413.0, 413.1, 413.9);  Asthma (493.x);  Arthritis (99.3, 274, 696.0, 695.4, 710.x, 711.x, 712.x, 714.x, 713.x, 715.x, 716.x, 719.x, 720.x, 721.x);  Stroke (430.x–438.x);  Chronic kidney disease (016.0x, 042.x, 095.4, 189.x, 223.x, 236.9x, 250.4x, 271.4, 274.1x, 403.x, 404.x, 440.1, 442.1, 446.21, 447.3x, 572.4, 580.x–589.x, 590.x, 591.x, 593.x, 642.1x, 646.2x, 753.x, 984.x) |
| Body weight loss | 260, 261, 262, 263.0, 263.1, 263.8, 263.9, 728.2, 780.94, 783.21, 783.3, 783.7, 799.4 |
| **Diagnostic codes used to identify comorbidities associated with T1D and T2D** | |
| Hypertension | 401.x, 402.x, 402.0x, 402.1x, 402.9x, 403.x, 403.0x, 403.1x, 403.9x, 404.x, 404.0x, 404.1x, 404.9, 404.9x, 405.x, 405.0x, 405.1, 405.1x, 405.9, 405.9x |
| Hyperlipidemia | 272.x |
| Acute coronary syndrome | 410.0x, 410.1, 410.1x, 410.2, 410.2x, 410.3, 410.3x, 410.4, 410.4x, 410.5, 410.5x, 410.6, 410.6x, 410.7, 410.7x, 410.8, 410.8x, 410.9, 410.9x, 411.x, 411.8x, 412, 413.x, 414.0, 414.x 414.0x, 414.1, 414.1x, 414.2 |
| Cardiac ischemia | 410.x, 411.x, 412, 413.x, 414.x |
| Myocardial infarction | 410.0x, 410.1, 410.1x, 410.2, 410.2x, 410.3, 410.3x, 410.4, 410.4x, 410.5, 410.5x, 410.6, 410.6x, 410.7, 410.7x, 410.8, 410.8x, 410.9, 410.9x, 412 |
| Peripheral artery disease | 440.x, 441.2x, 440.3, 440.3x, 441.x, 441.0x, 441.1x, 442.x, 442.8x, 443.x, 443.2x, 444.x, 444.0x, 444.2x, 444.8x, 447.x, 447.7x, 557.x |
| Abnormal blood chemistry | 790.6 |
| Chronic kidney disease stages | 585.3, 585.4, 585.5, 585.6 |
| Unstable angina | 411.x, 411.8x |
| Congestive heart failure | 428.x, 428.2x, 428.3x, 428.4x |
| Transient ischemic attack | 435.x |
| Ischemic stroke | 433.x1, 434.x1 |
| Microalbuminuria | 791.0 |
| Retinopathy | 249.x, 249.5x, 250.5, 250.5x, 362.x, 362.0x, 362.1, 362.1x, 362.2, 362.2x, |
| Carotid arterial disease | 433.x, 433.1x, 437.3, 442.81 |
| Left ventricular dysfunction | 428.1 |
| Left ventricular hypertrophy | 429.3 |

T1D, type 1 diabetes; T2D, type 2 diabetes.

| Table S2. Previous prescriptions by type of diabetes and by age group in previously diagnosed patients | | | | | | | | |
| --- | --- | --- | --- | --- | --- | --- | --- | --- |
|  | T1D | | | | T2D | | | Total T1D and T2D  N=345 |
|  | Age range in years | | | | Age range in years | | |  |
| Variable | <18  N=50 | 18-64  N=186 | ≥65  N=21 | Total  N=257 | 18-64  N=64 | ≥65  N=24 | Total  N=88 |  |
| Receiving prior insulin treatment | 46 (92.0) | 178 (95.7) | 21 (100) | 245 (95.3) | 60 (93.8) | 21 (87.5) | 81 (92.0) | 326 (94.5) |
| Basal insulin | 45 (90.0) | 177 (95.2) | 21 (100) | 243 (94.6) | 58 (90.6) | 20 (83.3) | 78 (88.6) | 321 (93.0) |
| -Degludec insulin | 3 (6.0) | 53 (28.5) | 11 (52.4) | 67 (26.1) | 11 (17.2) | 8 (33.3) | 19 (21.6% | 86 (24.9) |
| -Tresiba® | 3 (6.0) | 53 (28.5) | 11 (52.4) | 67 (26.1) | 11 (17.2) | 8 (33.3) | 19 (21.6) | 86 (24.9) |
| -Detemir insulin | 12 (24.0) | 19 (10.2) | 2 (9.5) | 33 (12.8) | 6 (9.4) | 4 (16.7) | 10 (11.4) | 43 (12.5) |
| -Levemir® | 12 (24.0) | 19 (10.2) | 2 (9.5) | 33 (12.8) | 6 (9.4) | 4 (16.7) | 10 (11.4) | 43 (12.5) |
| -Glargine insulin | 38 (76.0) | 167 (89.8) | 18 (85.7) | 223 (86.8) | 57 (89.1) | 19 (79.2) | 76 (86.4) | 299 (86.7) |
| -Abasaglar® | 22 (44.0) | 11 (5.9) | - | 33 (12.8) | 2 (3.1) | 1 (4.2) | 3 (3.4) | 36 (10.4) |
| -Lantus® | 17 (34.0) | 147 (79.0) | 18 (85.7) | 182 (70.8) | 48 (75.0) | 18 (75.0) | 66 (75.0) | 248 (71.9) |
| -Toujeo® | 1 (2.0) | 75 (40.3) | 11 (52.4) | 87 (33.9) | 28 (43.8) | 4 (16.7) | 32 (36.4) | 119 (34.5) |
| -Isophane insulin | 3 (6.0) | 7 (3.8) | 1 (4.8) | 11 (4.3) | 3 (4.7) | 1 (4.2) | 4 (4.5) | 15 (4.3) |
| -Humulin® NPH | 2 (4.0) | 1 (0.5) | 1 (4.8) | 4 (1.6) | - | - | - | 4 (1.2) |
| -Insulatard NPH® | 1 (2.0) | 6 (3.2) | - | 7 (2.7) | 3 (4.7) | 1 (4.2) | 4 (4.5) | 11 (3.2) |
| -Lispro Protamine insulin | - | 1 (0.5) | - | 1 (0.4) | - | - | - | 1 (0.3) |
| -Humalog® Basal | - | 1 (0.5) | - | 1 (0.4) | - | - | - | 1 (0.3) |
| Mixed insulin | - | 14 (7.5) | 2 (9.5) | 16 (6.2) | 11 (17.2) | 5 (20.8) | 16 (18.2) | 32 (9.3) |
| -Aspart Protamine insulin | - | 8 (4.3) | 1 (4.8) | 9 (3.5) | 7 (10.9) | 3 (12.5) | 10 (11.4) | 19 (5.5) |
| -NovoMix® | - | 8 (4.3) | 1 (4.8) | 9 (3.5) | 7 (10.9) | 3 (12.5) | 10 (11.4) | 19 (5.5) |
| -Human Isofanic insulin | - | 1 (0.5) | - | 1 (0.4) | - | - | - | 1 (0.3) |
| -Humulin® 30:70 | - | 1 (0.5) | - | 1 (0.4) | - | - | - | 1 (0.3) |
| -Lispro Protamine insulin | - | 5 (2.7) | 1 (4.8) | 6 (2.3) | 4 (6.3) | 2 (8.3) | 6 (6.8) | 12 (3.5) |
| -Humalog® Mix | - | 5 (2.7) | 1 (4.8) | 6 (2.3) | 4 (6.3) | 2 (8.3) | 6 (6.8) | 12 (3.5) |
| Rapid insulin | 43 (86.0) | 173 (93.0) | 20 (95.2) | 236 (91.8) | 51 (79.7) | 16 (66.7) | 67 (76.1) | 303 (87.8) |
| -Aspart insulin | 27 (54.0) | 124 (66.7) | 17 (81.0) | 168 (65.4) | 31 (48.4) | 10 (41.7) | 41 (46.6) | 209 (60.6) |
| -Fiasp® | - | 15 (8.1) | 2 (9.5) | 17 (6.6) | 4 (6.3) | - | 4 (4.5) | 21 (6.1) |
| -NovoRapid® | 27 (54.0) | 117 (62.9) | 16 (76.2) | 160 (62.3) | 30 (46.9) | 10 (41.7) | 40 (45.5) | 200 (58.0) |
| -Glulisine insulin | 6 (12.0) | 46 (24.7) | 7 (33.3) | 59 (23.0) | 15 (23.4) | 3 (12.5) | 18 (20.5) | 77 (22.3) |
| -Apidra® | 6 (12.0) | 46 (24.7) | 7 (33.3) | 59 (23.0) | 15 (23.4) | 3 (12.5) | 18 (20.5) | 77 (22.3) |
| -Human insulin | - | 3 (1.6) | 1 (4.8) | 4 (1.6) | 3 (4.7) | 1 (4.2) | 4 (4.5) | 8 (2.3) |
| -Actrapid® | - | 3 (1.6) | 1 (4.8) | 4 (1.6) | 3 (4.7) | 1 (4.2) | 4 (4.5) | 8 (2.3) |
| -Lispro insulin | 16 (32.0) | 37 (19.9) | 5 (23.8) | 58 (22.6) | 12 (18.8) | 6 (25.0) | 18 (20.5) | 76 (22.0) |
| -Humalog® 100 | 15 (30.0) | 36 (19.4) | 5 (23.8) | 56 (21.8) | 10 (15.6) | 6 (25.0) | 16 (18.2) | 72 (20.9) |
| -Humalog® 200 | 2 (4.0) | 3 (1.6) | - | 5 (1.9) | 2 (3.1) | - | 2 (2.3) | 7 (2.0) |
| Non-insulin antidiabetic treatment | - | - | - | - | 61 (95.3) | 23 (95.8) | 84 (95.5) | 84 (24.3) |
| -Metformin (alone/combined) | - | - | - | - | 48 (75.0) | 22 (91.7) | 70 (79.5) | 70 (20.3) |
| -Metformin (alone) | - | - | - | - | 44 (68.8) | 16 (66.7) | 60 (68.2) | 60 (17.4) |
| -DPP-IV | - | - | - | - | 11 (17.2) | 8 (33.3) | 19 (21.6) | 19 (5.5) |
| -Linagliptin | - | - | - | - | 4 (6.3) | 3 (12.5) | 7 (8.0) | 7 (2.0) |
| -Trajenta® | - | - | - | - | 4 (6.3) | 3 (12.5) | 7 (8.0) | 7 (2.0) |
| -Saxagliptin | - | - | - | - | 1 (1.6) | - | 1 (1.1) | 1 (0.3) |
| -Onglyza® | - | - | - | - | 1 (1.6) | - | 1 (1.1) | 1 (0.3) |
| -Sitagliptin | - | - | - | - | 5 (7.8) | 3 (12.5) | 8 (9.1) | 8 (2.3) |
| -Januvia® | - | - | - | - | 2 (3.1) | 2 (8.3) | 4 (4.5) | 4 (1.2) |
| -Sitagliptin | - | - | - | - | 3 (4.7) | - | 3 (3.4) | 3 (0.9) |
| -Tesavel® | - | - | - | - | 1 (1.6) | - | 1 (1.1) | 1 (0.3) |
| -Xelevia® | - | - | - | - | 2 (3.1) | 1 (4.2) | 3 (3.4) | 3 (0.9) |
| -Vildagliptin | - | - | - | - | 3 (4.7) | 3 (12.5) | 6 (6.8) | 6 (1.7) |
| -Galvus® | - | - | - | - | 1 (1.6) | 1 (4.2) | 2 (2.3) | 2 (0.6) |
| -Jalra® | - | - | - | - | - | 1 (4.2) | 1 (1.1) | 1 (0.3) |
| -Vildagliptin PPA | - | - | - | - | 2 (3.1) | 2 (8.3) | 4 (4.5) | 4 (1.2) |
| -DPP-IV+Glitazone | - | - | - | - | 1 (1.6) | - | 1 (1.1) | 1 (0.3) |
| -Alogliptin+Pioglitazone | - | - | - | - | 1 (1.6) | - | 1 (1.1) | 1 (0.3) |
| -Incresync® | - | - | - | - | 1 (1.6) | - | 1 (1.1) | 1 (0.3) |
| -DPP-IV+Metformin | - | - | - | - | 19 (29.7) | 13 (54.2) | 32 (36.4) | 32 (9.3) |
| -Linagliptin+Metformin | - | - | - | - | 2 (3.1) | 3 (12.5) | 5 (5.7) | 5 (1.4) |
| -Jentadueto® | - | - | - | - | 2 (3.1) | 3 (12.5) | 5 (5.7) | 5 (1.4) |
| -Sitagliptin+Metformin | - | - | - | - | 10 (15.6) | 6 (25.0) | 16 (18.2) | 16 (4.6) |
| -Efficib® | - | - | - | - | 2 (3.1) | 1 (4.2) | 3 (3.4) | 3 (0.9) |
| -Janumet® | - | - | - | - | 3 (4.7) | 3 (12.5) | 6 (6.8) | 6 (1.7) |
| -Ristfor® | - | - | - | - | 1 (1.6) | - | 1 (1.1) | 1 (0.3) |
| -Sitagliptin | - | - | - | - | 6 (9.4) | 1 (4.2) | 7 (8.0) | 7 (2.0) |
| -Velmetia® | - | - | - | - | 1 (1.6) | 1 (4.2) | 2 (2.3) | 2 (0.6) |
| -Vildagliptin+Metformin | - | - | - | - | 8 (12.5) | 5 (20.8) | 13 (14.8) | 13 (3.8) |
| -Eucreas® | - | - | - | - | 3 (4.7) | 2 (8.3) | 5 (5.7) | 5 (1.4) |
| -Vildagliptin | - | - | - | - | 6 (9.4) | 4 (16.7) | 10 (11.4) | 10 (2.9) |
| -SGLT2 inhibitors | - | - | - | - | 15 (23.4) | 5 (20.8) | 20 (22.7) | 20 (5.8) |
| -Canagliflozin | - | - | - | - | 2 (3.1) | 1 (4.2) | 3 (3.4) | 3 (0.9) |
| -Invokana® | - | - | - | - | 2 (3.1) | 1 (4.2) | 3 (3.4) | 3 (0.9) |
| -Dapagliflozin | - | - | - | - | 6 (9.4) | 1 (4.2) | 7 (8.0) | 7 (2.0) |
| -Forxiga® | - | - | - | - | 6 (9.4) | 1 (4.2) | 7 (8.0) | 7 (2.0) |
| -Empagliflozin | - | - | - | - | 7 (10.9) | 3 (12.5) | 10 (11.4) | 10 (2.9) |
| -Jardiance® | - | - | - | - | 7 (10.9) | 3 (12.5) | 10 (11.4) | 10 (2.9) |
| -SGLT2+Metformin | - | - | - | - | 5 (7.8) | 1 (4.2) | 6 (6.8) | 6 (1.7) |
| -Dapagliflozin+Metformin | - | - | - | - | 3 (4.7) | - | 3 (3.4) | 3 (0.9) |
| -Xigduo® | - | - | - | - | 3 (4.7) | - | 3 (3.4) | 3 (0.9) |
| -Empagliflozin+Metformin | - | - | - | - | 2 (3.1) | 1 (4.2) | 3 (3.4) | 3 (0.9) |
| -Synjardy® | - | - | - | - | 2 (3.1) | 1 (4.2) | 3 (3.4) | 3 (0.9) |
| -GLP-1 | - | - | - | - | 3 (4.7) | - | 3 (3.4) | 3 (0.9) |
| -Dulaglutide | - | - | - | - | 1 (1.6) | - | 1 (1.1) | 1 (0.3) |
| -Trulicity® | - | - | - | - | 1 (1.6) | - | 1 (1.1) | 1 (0.3) |
| -Exenatide | - | - | - | - | 2 (3.1) | - | 2 (2.3) | 2 (0.6) |
| -Bydureon® | - | - | - | - | 2 (3.1) | - | 2 (2.3) | 2 (0.6) |
| -Alpha Glucosidase Inhibitors | - | - | - | - | 1 (1.6) | - | 1 (1.1) | 1 (0.3) |
| -Acarbose | - | - | - | - | 1 (1.6) | - | 1 (1.1) | 1 (0.3) |
| -Glucobay® | - | - | - | - | 1 (1.6) | - | 1 (1.1) | 1 (0.3) |
| -Prandial Glucose Regulators | - | - | - | - | 4 (6.3) | 4 (16.7) | 8 (9.1) | 8 (2.3) |
| -Repaglinide | - | - | - | - | 4 (6.3) | 4 (16.7) | 8 (9.1) | 8 (2.3) |
| -Novonorm® | - | - | - | - | 2 (3.1) | 2 (8.3) | 4 (4.5) | 4 (1.2) |
| -Repaglinide | - | - | - | - | 3 (4.7) | 3 (12.5) | 6 (6.8) | 6 (1.7) |
| -Sulfonylureas | - | - | - | - | 12 (18.8) | 7 (29.2) | 19 (21.6) | 19 (5.5) |
| -Glibenclamide | - | - | - | - | 2 (3.1) | 3 (12.5) | 5 (5.7) | 5 (1.4) |
| -Daonil® | - | - | - | - | 2 (3.1) | - | 2 (2.3) | 2 (0.6) |
| -Glibenclamide | - | - | - | - | - | 3 (12.5) | 3 (3.4) | 3 (0.9) |
| -Gliclazide | - | - | - | - | 6 (9.4) | 2 (8.3) | 8 (9.1) | 8 (2.3) |
| -Diamicron® | - | - | - | - | 1 (1.6) | - | 1 (1.1) | 1 (0.3) |
| -Gliclazide | - | - | - | - | 6 (9.4) | 2 (8.3) | 8 (9.1) | 8 (2.3) |
| -Glimepiride | - | - | - | - | 3 (4.7) | 3 (12.5) | 6 (6.8) | 6 (1.7) |
| -Amaryl® | - | - | - | - | - | 1 (4.2) | 1 (1.1) | 1 (0.3) |
| -Glimepiride | - | - | - | - | 3 (4.7) | 3 (12.5) | 6 (6.8) | 6 (1.7) |
| -Glipizide | - | - | - | - | 1 (1.6) | - | 1 (1.1) | 1 (0.3) |
| -Glipizide | - | - | - | - | 1 (1.6) | - | 1 (1.1) | 1 (0.3) |
| Pre-Jr KwikPen figures were calculated excluding newly diagnosed patients. This table includes the number and percentage (%) of patients who received any of the listed treatments, with it being possible that the same patient received more than one treatment during the period, as it was a multiresponse option. Limitation: Some concomitant prescriptions may have fallen outside this time window. Therefore, some treatments (e.g., basal insulin) may be underrepresented. DPP-IV, dipeptidyl peptidase IV; GLP-1, glucagon-like peptide-1; Jr KwikPen, Junior KwikPen; NPH, neutral protamine hagedorn; SGLT2, sodium-glucose co-transporter 2; T1D, type 1 diabetes; T2D, type 2 diabetes. | | | | | | | | |

| Table S3. Concomitant antidiabetic prescriptions (including Jr KwikPen) received within 60 days from Jr KwikPen treatment initiation overall, by type of diabetes and by age group in the overall sample | | | | | | | | |
| --- | --- | --- | --- | --- | --- | --- | --- | --- |
|  | T1D | | | | T2D | | | Total T1D and T2D  N=416 |
|  | Age range in years | | | | Age range in years | | |  |
| Variable | <18  N=107 | 18-64  N=194 | ≥65  N=25 | Total  N=326 | 18-64  N=64 | ≥65  N=26 | Total  N=90 |  |
| Receiving post-insulin treatment | 107 (100) | 194 (100) | 25 (100) | 326 (100) | 64 (100) | 26 (100) | 90 (100) | 416 (100) |
| -Basal insulin | 81 (75.7) | 114 (58.8) | 13 (52.0) | 208 (63.8) | 44 (68.8) | 17 (65.4) | 61 (67.8) | 269 (64.7) |
| -Degludec insulin | 4 (3.7) | 31 (16.0) | 2 (8.0) | 37 (11.3) | 8 (12.5) | 4 (15.4) | 12 (13.3) | 49 (11.8) |
| -Tresiba® | 4 (3.7) | 31 (16.0) | 2 (8.0) | 37 (11.3) | 8 (12.5) | 4 (15.4) | 12 (13.3) | 49 (11.8) |
| -Detemir insulin | 27 (25.2) | 4 (2.1) | 1 (4.0) | 32 (9.8) | 1 (1.6) | 2 (7.7) | 3 (3.3) | 35 (8.4) |
| -Levemir® | 27 (25.2) | 4 (2.1) | 1 (4.0) | 32 (9.8) | 1 (1.6) | 2 (7.7) | 3 (3.3) | 35 (8.4) |
| -Glargine insulin | 50 (46.7) | 80 (41.2) | 10 (40.0) | 140 (42.9) | 36 (56.3) | 11 (42.3) | 47 (52.2) | 187 (45.0) |
| -Abasaglar® | 38 (35.5) | 5 (2.6) | 1 (4.0) | 44 (13.5) | 1 (1.6) | 1 (3.8) | 2 (2.2) | 46 (11.1) |
| -Lantus® | 10 (9.3) | 25 (12.9) | 4 (16.0) | 39 (12.0) | 12 (18.8) | 6 (23.1) | 18 (20.0) | 57 (13.7) |
| -Toujeo® | 2 (1.9) | 51 (26.3) | 5 (20.0) | 58 (17.8) | 24 (37.5) | 4 (15.4) | 28 (31.1) | 86 (20.7) |
| -Mixed insulin | - | 1 (0.5) | - | 1 (0.3) | - | - | - | 1 (0.2) |
| -Lispro Protamine insulin | - | 1 (0.5) | - | 1 (0.3) | - | - | - | 1 (0.2) |
| -Humalog® Mix | - | 1 (0.5) | - | 1 (0.3) | - | - | - | 1 (0.2) |
| -Rapid insulin | 107 (100) | 194 (100) | 25 (100) | 326 (100) | 64 (100) | 26 (100) | 90 (100) | 416 (100) |
| -Aspart insulin | 3 (2.8) | 8 (4.1) | 1 (4.0) | 12 (3.7) | 1 (1.6) | - | 1 (1.1) | 13 (3.1) |
| -Fiasp® | - | 2 (1.0) | 1 (4.0) | 3 (0.9) | - | - | - | 3 (0.7) |
| -NovoRapid® | 3 (2.8) | 6 (3.1) | - | 9 (2.8) | 1 (1.6) | - | 1 (1.1) | 10 (2.4) |
| -Glulisine insulin | - | 1 (0.5) | 1 (4.0) | 2 (0.6) | - | - | - | 2 (0.5) |
| -Apidra® | - | 1 (0.5) | 1 (4.0) | 2 (0.6) | - | - | - | 2 (0.5) |
| -Lispro insulin | 107 (100) | 194 (100) | 25 (100) | 326 (100) | 64 (100) | 26 (100) | 90 (100) | 416 (100) |
| -Humalog® 100^a^ | 107 (100) | 194 (100) | 25 (100) | 326 (100) | 64 (100) | 26 (100) | 90 (100) | 416 (100) |
| -Humalog® 200 | - | 1 (0.5) | - | 1 (0.3) | 1 (1.6) | - | 1 (1.1) | 2 (0.5) |
| -Receiving post non-insulin antidiabetic prescription | - | - | - | - | 33 (51.6) | 17 (65.4) | 50 (55.6) | 50 (12.0) |
| -Metformin (alone/combined) | - | - | - | - | 26 (40.6) | 15 (57.7) | 41 (45.6) | 41 (9.9) |
| -Metformin (alone) | - | - | - | - | 16 (25.0) | 9 (34.6) | 25 (27.8) | 25 (6.0) |
| -DPP-IV | - | - | - | - | 3 (4.7) | 2 (7.7) | 5 (5.6) | 5 (1.2) |
| -Linagliptin | - | - | - | - | 1 (1.6) | 1 (3.8) | 2 (2.2) | 2 (0.5) |
| -Trajenta® | - | - | - | - | 1 (1.6) | 1 (3.8) | 2 (2.2) | 2 (0.5) |
| -Sitagliptin | - | - | - | - | 2 (3.1) | 1 (3.8) | 3 (3.3) | 3 (0.7) |
| -Sitagliptin | - | - | - | - | 2 (3.1) | 1 (3.8) | 3 (3.3) | 3 (0.7) |
| -DPP-IV+Metformin | - | - | - | - | 7 (10.9) | 7 (26.9) | 14 (15.6) | 14 (3.4) |
| -Linagliptin+Metformin | - | - | - | - | - | 2 (7.7) | 2 (2.2) | 2 (0.5) |
| -Jentadueto® | - | - | - | - | - | 2 (7.7) | 2 (2.2) | 2 (0.5) |
| -Sitagliptin+Metformin | - | - | - | - | 6 (9.4) | 3 (11.5) | 9 (10.0) | 9 (2.2) |
| -Efficib® | - | - | - | - | 2 (3.1) | - | 2 (2.2) | 2 (0.5) |
| -Janumet® | - | - | - | - | 1 (1.6) | 2 (7.7) | 3 (3.3) | 3 (0.7) |
| -Ristfor® | - | - | - | - | 1 (1.6) | - | 1 (1.1) | 1 (0.2) |
| -Sitagliptin | - | - | - | - | 1 (1.6) | 1 (3.8) | 2 (2.2) | 2 (0.5) |
| -Velmetia® | - | - | - | - | 1 (1.6) | - | 1 (1.1) | 1 (0.2) |
| -Vildagliptin+Metformin | - | - | - | - | 1 (1.6) | 2 (7.7) | 3 (3.3) | 3 (0.7) |
| -Eucreas® | - | - | - | - | 1 (1.6) | - | 1 (1.1) | 1 (0.2) |
| -Vildagliptin | - | - | - | - | - | 2 (7.7) | 2 (2.2) | 2 (0.5) |
| -SGLT2 | - | - | - | - | 5 (7.8) | 2 (7.7) | 7 (7.8) | 7 (1.7) |
| -Canagliflozin | - | - | - | - | - | 1 (3.8) | 1 (1.1) | 1 (0.2) |
| -Invokana® | - | - | - | - | - | 1 (3.8) | 1 (1.1) | 1 (0.2) |
| -Dapagliflozin | - | - | - | - | 3 (4.7) | - | 3 (3.3) | 3 (0.7) |
| -Forxiga® | - | - | - | - | 3 (4.7) | - | 3 (3.3) | 3 (0.7) |
| -Empagliflozin | - | - | - | - | 2 (3.1) | 1 (3.8) | 3 (3.3) | 3 (0.7) |
| -Jardiance® | - | - | - | - | 2 (3.1) | 1 (3.8) | 3 (3.3) | 3 (0.7) |
| -SGLT2+Metformin | - | - | - | - | 3 (4.7) | - | 3 (3.3) | 3 (0.7) |
| -Dapagliflozin+Metformin | - | - | - | - | 2 (3.1) | - | 2 (2.2) | 2 (0.5) |
| -Xigduo® | - | - | - | - | 2 (3.1) | - | 2 (2.2) | 2 (0.5) |
| -Empagliflozin+Metformin | - | - | - | - | 1 (1.6) | - | 1 (1.1) | 1 (0.2) |
| -Synjardy® | - | - | - | - | 1 (1.6) | - | 1 (1.1) | 1 (0.2) |
| -GLP-1 | - | - | - | - | 3 (4.7) | - | 3 (3.3) | 3 (0.7) |
| -Dulaglutide | - | - | - | - | 1 (1.6) | - | 1 (1.1) | 1 (0.2) |
| -Trulicity® | - | - | - | - | 1 (1.6) | - | 1 (1.1) | 1 (0.2) |
| -Exenatide | - | - | - | - | 1 (1.6) | - | 1 (1.1) | 1 (0.2) |
| -Bydureon® | - | - | - | - | 1 (1.6) | - | 1 (1.1) | 1 (0.2) |
| -Semaglutide | - | - | - | - | 1 (1.6) | - | 1 (1.1) | 1 (0.2) |
| -Ozempic® | - | - | - | - | 1 (1.6) | - | 1 (1.1) | 1 (0.2) |
| -Sulfonylureas | - | - | - | - | 1 (1.6) | - | 1 (1.1) | 1 (0.2) |
| -Gliclazide | - | - | - | - | 1 (1.6) | - | 1 (1.1) | 1 (0.2) |
| -Gliclazide | - | - | - | - | 1 (1.6) | - | 1 (1.1) | 1 (0.2) |
| Post-Jr KwikPen figures were calculated using the total population, including newly diagnosed patients (N=416). ^a^Includes Humalog Junior. Limitation: Some concomitant prescriptions may have fallen outside this time window. Therefore, some treatments (e.g., basal insulin) may be underrepresented. The table includes the number and percentage (%) of patients who received any of the listed treatments, with it being possible that the same patient received more than one treatment during the period, as it was a multiresponse option. DPP-IV, dipeptidyl peptidase IV; GLP-1, Glucagon-like peptide-1; Jr KwikPen, Junior KwikPen; NPH, neutral protamine hagedorn; SGLT2, sodium-glucose co-transporter 2; T1D, type 1 diabetes; T2D, type 2 diabetes. | | | | | | | | |

| Table S4. Mean daily dose of insulin prescribed within the 90 days before and within the 60 days after Jr KwikPen treatment initiation | | | | | | | | |
| --- | --- | --- | --- | --- | --- | --- | --- | --- |
|  | T1D | | | | T2D | | | Total T1D and T2D |
|  | Age range in years | | | | Age range in years | | |  |
| Variable | <18 | 18-64 | ≥65 | Total | 18-64 | ≥65 | Total |  |
| Mean daily dose of insulin prescribed within the 90 days before Jr KwikPen treatment initiation | | | | | | | | |
| Mean daily dose of basal insulin prescribed within the 90 days before the index date (UI/day) | N=30 | N=120 | N=15 | N=165 | N=41 | N=13 | N=54 | N=219 |
|  | 19.78 (7.58) | 26.08 (8.91) | 22.60 (11.36) | 24.62 (9.22) | 29.05 (11.14) | 30.08 (19.05) | 29.30 (13.27) | 25.77 (10.53) |
| Mean daily dose of rapid insulin prescribed within the 90 days before the index date (UI/day) | N=32 | N=86 | N=10 | N=128 | N=26 | N=8 | N=34 | N=162 |
|  | 36.80 (22.58) | 33.05 (14.75) | 21.50 (8.93) | 33.09 (17.03) | 30.87 (19.62) | 18.75 (7.98) | 28.01 (18.23) | 32.02 (17.35) |
| Mean daily dose of mixed insulin prescribed within the 90 days before the index date (UI/day) | - | N=2 | - | N=2 | N=1 | N=1 | N=2 | N=4 |
|  | - | 52.00 (11.31) | - | 52.00 (11.31) | 45.00 (.) | 28.00 (.) | 36.50 (12.02) | 44.25 (13.07) |
| Mean total daily dose of insulin prescribed within the 90 days before the index date (UI/day) | N=36 | N=142 | N=17 | N=195 | N=49 | N=15 | N=64 | N=259 |
|  | 49.19 (25.11) | 42.79 (23.43) | 32.59 (18.09) | 43.08 (23.59) | 41.60 (22.67) | 37.93 (18.09) | 40.74 (21.61) | 42.50 (23.10) |
| Mean daily dose of insulin prescribed within the 60 days after Jr KwikPen treatment initiation | | | | | | | | |
| Daily dose (UI/day) of basal insulin prescribed at index and within the 60 days after the index date (mean of all prescriptions in the period) | N=81 | N=113 | N=13 | N=207 | N=44 | N=17 | N=61 | N=268 |
|  | 13.75 (7.16) | 25.42 (9.61) | 21.62 (11.63) | 20.62 (10.45) | 28.60 (11.68) | 29.24 (19.42) | 28.78 (14.09) | 22.47 (11.86) |
| Daily dose (UI/day) of rapid insulin prescribed at index and within the 60 days after the index date (mean of all prescriptions in the period) | N=107 | N=194 | N=25 | N=326 | N=64 | N=26 | N=90 | N=416 |
|  | 26.49 (19.01) | 28.23 (15.86) | 23.21 (15.63) | 27.27 (16.95) | 26.27 (16.15) | 16.85 (6.58) | 23.55 (14.67) | 26.47 (16.54) |
| Daily dose (UI/day) of mixed insulin prescribed at index and within the 60 days after the index date (mean of all prescriptions in the period) | - | N=1 | - | N=1 | - | - | - | N=1 |
|  | - | 44.00 (.) | - | 44.00 (.) | - | - | - | 44.00 (.) |
| Total daily dose (UI/day) of insulin prescribed at index and within the 60 days after the index date (sum of mean of all prescriptions of insulin in the period) | N=107 | N=194 | N=25 | N=326 | N=64 | N=26 | N=90 | N=416 |
|  | 36.90 (21.75) | 43.26 (21.85) | 34.45 (19.46) | 40.50 (21.85) | 45.94 (25.50) | 35.96 (21.21) | 43.06 (24.64) | 41.05 (22.48) |

The N’s shown here represent the number of patients with prescriptions available. Mean doses were calculated based on the number of patients with valid doses. Jr KwikPen, Junior KwikPen; T1D, type 1 diabetes; T2D, type 2 diabetes.
